# Dynamics of leukocyte populations, immune-regulatory cytokines, and biochemical parameters in wild boar and domestic pigs experimentally infected with a virulent African swine fever virus genotype II strain

**DOI:** 10.3389/fimmu.2026.1751646

**Published:** 2026-03-24

**Authors:** Giulia Franzoni, Fabian Zhi Xiang Lean, Emanuela Giaconi, Giuseppe Tedde, Susanna Zinellu, Paola Nicolussi, Mireille Le Dimna, Marie-Frédérique Le Potier, Elliot Steedman, Helen Rachel Crooke, Cecilia Righi, Stefano Petrini, Noemí Rayón, Dolores Gavier-Widen, Alejandro Núñez, Pedro Jose Sanchez-Cordon

**Affiliations:** 1 Department of Animal Health, Istituto Zooprofilattico Sperimentale della Sardegna, Sassari, Italy; 2 Pathology and Animal Sciences Department, Animal and Plant Health Agency (APHA-Weybridge), New Haw, United Kingdom; 3 Department of Clinical Diagnostic, Istituto Zooprofilattico Sperimentale della Sardegna, Sassari, Italy; 4 French Agency for Food, Environmental and Occupational Health & Safety (Anses), Ploufragan-Plouzané-Niort laboratory Swine Virology, Innovation and Genomics Unit, Ploufragan, France; 5 Department of Virology, Animal and Plant Health Agency, (APHA-Weybridge), New Haw, United Kingdom; 6 National Reference Centre for Pestiviruses and Asfivirus, Istituto Zooprofilattico Sperimentale Umbria-Marche “Togo Rosati”, Perugia, Italy; 7 Department of Veterinary Pathology, Alfonso X “El Sabio” University (UAX), Madrid, Spain; 8 Department of Pathology and Wildlife Diseases, Swedish Veterinary Agency (SVA), Uppsala, Sweden; 9 Department of Biomedical Sciences and Veterinary Public Health, Swedish University of Agricultural Sciences (SLU), Uppsala, Sweden; 10 Centro de Investigación en Sanidad Animal (CISA), Instituto Nacional de Investigación y Tecnología Agraria y Alimentaria (INIA), Consejo Superior de Investigaciones Científicas (CSIC), Madrid, Spain

**Keywords:** African swine fever virus, biochemical parameters, cytokines, leukocytes, pigs, wild boar

## Abstract

African Swine Fever Virus (ASFV) is the causative agent of devastating disease affecting domestic and wild pigs globally. A previous study of the intranasal inoculation of domestic pigs (DP) and wild boar (WB) with the ASFV genotype II strain “Armenia 2007” demonstrated distinct disease outcomes. This study aims to compare the leukocyte, cytokine and biochemical profiles in experimentally infected DP and WB. Blood and serum samples were collected before infection (day 0), from animals euthanized in groups of six (comprising 3 DP and 3 WB) on days 1, 2, 3 and 5 post infection (pi) and from animals that reached a humane endpoint. Both DP and WB developed severe lymphopenia, occurring earlier in WB. Inflammatory response occurred earlier in WB, as evident from day 3 pi by the increased levels of TNF, followed by IL-6 and, to a lesser extent, IL-1β. IL-8 concentrations only increased in some WB, but not in DP. No modulation of Th1-associated cytokines (IFN-γ, IL-12 and IL-18) was detected in DP, whereas WB had a moderate increase in IL-12 and IFN-γ levels from day 5 pi, which peaked at humane endpoint. C-reactive protein levels increased in concomitant with the release of pro-inflammatory cytokines, as early as day 5 pi in WB, reaching its maximum in both DP and WB at the humane endpoint. A delayed but significant increase in the levels of anti-inflammatory mediators IL-1Ra and IL-10 was observed in both groups, but earlier in some WB from day 5 pi. Biochemical analysis revealed potential perturbations of the liver function in both subspecies, characterized by changes in serum AST and triglycerides levels, in addition to renal alterations in DP evidenced by changes in creatinine and urea levels. These findings underscore earlier immune activation in WB, potentially contributing to the different subspecies-specific disease outcomes following ASFV inoculation.

## 1 Introduction

African swine fever (ASF) is an infectious hemorrhagic disease of suids which affects both domestic pigs (DP) and wild boar (WB) of various breeds and ages, resulting in substantial lethality rates exceeding 90% among susceptible animals (1). Since highly virulent genotype II strains entered Georgia from south-eastern Africa in 2007 (2), ASF has spread rapidly around the world, affecting over 50 countries in Europe, Asia, the Caribbean, and Africa (3), constituting one of the major threats to the global swine industry (1). The etiological agent of ASF, the African swine fever virus (ASFV), is a large, complex, double-stranded DNA virus belonging to the Asfarviridae family within the Asfivirus genus (4). ASF viruses are classified into 24 genotypes based on the partial sequence of the B646L gene (4). All genotypes are present in Africa, whereas genotypes I and II have mainly circulated in Europe and Asia in recent decades (1, 5). Recently, highly virulent recombinant ASFV Genotype I/II strains have also been detected in China and Vietnam (6, 7).

The lack of available vaccines or treatments is hampering efforts to control the disease. Developing vaccines against ASF is challenging, so the disease is currently controlled by implementing rigorous biosecurity measures and culling all susceptible pigs on infected premises (8, 9). Therefore, there is an urgent need to improve our understanding of the immunopathogenic mechanisms behind ASFV infection in order to design safer and more efficient vaccines (10, 11). It is well known that, in acute lethal forms of ASF induced by highly virulent strains, the virus is capable of evading the host’s innate immune response. This is achieved by encoding a series of viral proteins that interfere with the induction of interferons, the secretion of immunomodulatory cytokines, apoptosis mechanisms, or antigen presentation processes, thereby altering early innate and adaptive immune responses (12, 13). Previous studies have also shown that infection with virulent strains of ASFV often leads to an exacerbated immune response characterized by the excessive release of pro-inflammatory cytokines, known as a ‘cytokine storm’. This can result in severe clinical signs, widespread inflammation, severe tissue damage, lymphopenia and an immunosuppressed state due to the depletion of lymphoid organs. Other studies have suggested a correlation between virus-specific IFN-γ-producing cells and disease protection (reviewed by 10).

Traditionally, studies on the immuno-pathogenic mechanisms of ASFV *in vivo* have mainly focused on DP, whereas research in this area is less extensive for WB (14–16). WB play an important role in the geographical spread of the disease in Europe and Asia, as they act as reservoirs for the virus, hindering eradication efforts (17, 18). Therefore, it is essential to understand such mechanisms in these subspecies, and to highlight any possible differences with DP. Having an accurate picture of the immune dysregulation mechanisms that operate in the early stages of deadly forms of ASF, in both DP and WB, is critical for developing and optimizing control strategies, such as vaccines and vaccination programmes, which should be tailored specifically to each host population (11, 19, 20).

Previous *in vivo* studies have shown that WB are more susceptible to ASFV infection than DP (21–24), and that WB had an earlier onset of clinical signs (24, 25). Differences in immune responses to infection with the virulent Armenia08 have also been described, with activated CD8^+^ T and γδ T cells detected in the WB, but not DP (25). Further, regulatory T cells (Tregs) increased earlier and at higher frequencies in WB compared to DP, which was correlated with the greater severity and lethality of the disease observed in WB (25). Differences between DP and WB were also described in experiments with the moderately virulent strain Estonia2018, in which no substantial γδ T cell response was observed in DP following infection. However, there was a significant bias towards γδ T cell responses in the WB, which was correlated with greater disease severity and lethality following ASFV infection (26).

Our previous comparative studies also demonstrated that WB were more susceptible than DP following intranasal infection with “Armenia 2007” ASFV strain. WB had shorter incubation periods, an earlier onset of clinical signs, viremia, and frequent macroscopic hemorrhagic lesions compared to DP, and reached a humane endpoint 3 days earlier than DP despite showing less severe macroscopic lesions (27). To improve the understanding of these disease differences, as well as the impact of the virus on the immune systems, hematological, immunological and biochemical assays were performed on blood and serum samples taken from the experimentally infected animals at different timepoints. The data generated will provide a clearer profile of the early immunopathogenic mechanisms induced by ASFV in these suid subspecies.

## 2 Materials and methods

### 2.1 Animals, experimental design and samples collection

**Figure 1:**
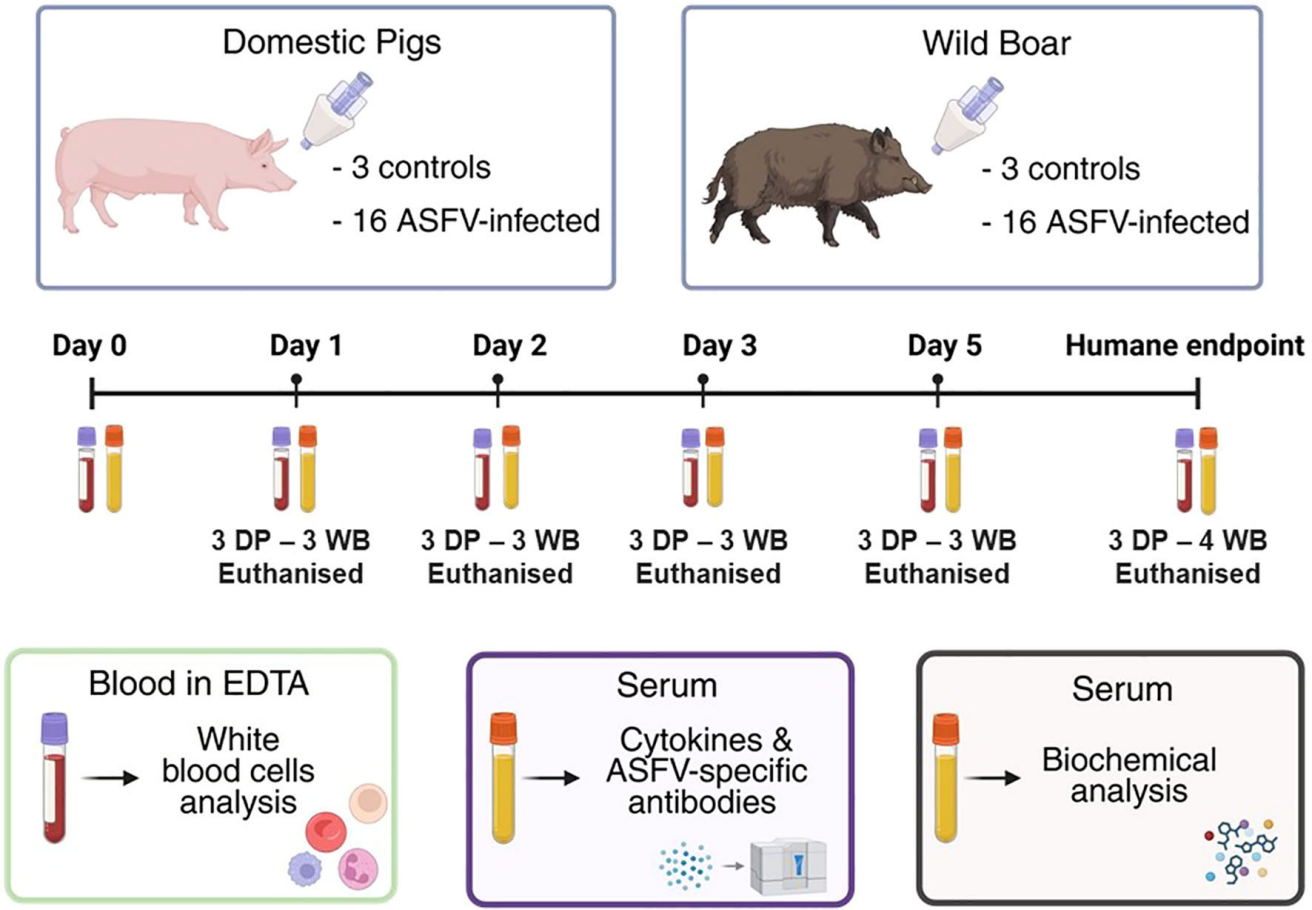
Overview of experimental design, sampling and analyses. Sixteen domestic pigs (DP) and sixteen wild boar (WB) were intranasally inoculated (IN) with the ASFV genotype II strain “Armenia 2007”, with three DP and three WB were euthanized on each time points of days 1, 2, 3, and 5 post-infection, and all the remaining WB and DP were euthanized at humane endpoints on day 6 and day 9 pi, respectively. Two control groups (three DP or three WB) were mock-inoculated. (pi), and euthanized at the end of the experiment on day 12 pi. Blood and serum samples were collected from all ASFV-infected and mock inoculated animals before inoculation (day 0) and at endpoints, and additionally at day 8 pi for mock inoculated groups. Fresh EDTA blood samples were used for hematology; serum samples used for cytokine, antibody responses, and biochemical analyses. Image created with Biorender.com (accessed on 10^th^ October 2025).

Details of the experimental design have been described previously (27) and further outlined in the schematic **Figure 1**. Briefly, thirty-eight animals (19 pigs aged 10–12 weeks and 19 wild boar aged 16–18 weeks) of both sexes were randomly allocated into four groups consisting of 8 animals each (either wild boar or pigs), which were assigned as the infected groups. Two further groups, consisting of either 3 pigs or 3 wild boar, were assigned as controls. Before inoculation, the animals in the infected groups were randomly assigned to predetermined timepoints at which they would be euthanized following infection. The animals were sedated by injecting a mixture of Zoletil and Domitor intramuscularly (0.5 mL of each product per 10 kg of body weight) and then intranasally inoculated (1 ml containing 10^4^ HAD_50_) with the ASFV virulent strain “Armenia 2007” (genotype II). On days 1, 2, 3, and 5 post-infection (pi), six inoculated animals (3 pigs and 3 wild boar) were sedated as described above and then euthanized by administering Pentoject intravenously (4 mL per 10 kg of body weight). The remaining inoculated animals (4 pigs and 4 wild boar) were euthanized upon reaching predetermined humane endpoints at day 6 (all remaining wild boar) and day 9 pi (all remaining pigs). Pig DP#38 did not become viremic or reach the humane endpoint, but was euthanized with its stablemates to prevent single housing. This animal was excluded from the present study. The control animals (n=6) were mock-infected with RPMI cell culture medium (Gibco) instead of the viral inoculum and euthanized at the conclusion of the experiment (day 12 pi).

Animals were clinically monitored daily. Additionally, EDTA blood and serum samples were collected from the anterior vena cava throughout the study to evaluate the impact of the virus on the immune systems and health status of DP and WB. Samples were taken from all ASFV-infected animals before inoculation (day 0) and at scheduled euthanasia timepoints on days 1, 2, 3, and 5 pi, as well as at the humane endpoint (day 6 pi for WB and day 9 pi for DP). Blood and serum samples were also collected from control animals that were mock-infected before mock infection (day 0) and on day 8 pi. Fresh EDTA blood samples were used for hematological analysis (section 2.2) while serum samples were frozen at -80°C for subsequent analysis of cytokine dynamics, antibody response and biochemical parameters indicative of tissue damage (section 2.4).

### 2.2 White blood cells analysis

White blood cell (WBC) count, including the main leukocytes subsets (granulocytes, lymphocytes, and monocytes) was performed on fresh EDTA blood samples taken from control animals and animals infected with the ASFV “Armenia 2007” strain. The total number of leukocytes (WBC) and the main leukocytes subsets were evaluated by immunostaining with anti-CD45-FITC (Clone K252.1E4, Bio-Rad Antibodies, Kidlington, UK) and analyzed by flow cytometry using a MACSQuant, analyzer (Miltenyi Biotech, Bisley UK). The total counts were plotted against the y-axis (1 × 10^6^/mL).

### 2.3 Impact of heat treatment on cytokine levels and biochemical parameters in serum samples from domestic pigs used for routine laboratory diagnostics

To evaluate the potential effect of heat treatment procedures, applied to reduce risk and facilitate sample transport, on cytokine levels and biochemical parameters (28–30), serum samples were collected from 30 domestic pigs undergoing routine diagnostic testing at the Virology Laboratory (Istituto Zooprofilattico Sperimentale della Sardegna, Italy). These serum samples were divided into two sets. One set was left untreated, while the other was treated at 56 °C for 30 minutes. The samples were then stored at -80 °C until they were analyzed.

Levels of IL-1α, IL-1β, IL-6, IL-8, IL-12, IL-18, and IL-1Ra were assessed in these serum samples after heat treatment (**Supplementary Figure S1**) using a Porcine Cytokine/Chemokine Magnetic Bead Panel Multiplex assay (Merck Milli-pore, Darmstadt, Germany) and a Bioplex MAGPIX Multiplex Reader (Bio-Rad, Hercu-les, CA, USA), according to the manufacturers´ instructions (31). All samples were tested in duplicate (2 technical replicates). In addition, serum levels of different biochemical parameters (CPK, ALP, GGT, C-reactive protein, AST, ALT, triglycerides, cholesterol, creatinine, calcium, urea, glucose, total protein and albumin; **Supplementary Figure S2**) were also evaluated using a Dimension RXL chemistry analyzer (Siemens) (32).

### 2.4 Evaluation of cytokine levels, antibody responses and biochemical parameters in control animals and animals infected with the ASFV “Armenia 2007” strain

Aliquots of serum samples collected from DP and WB during the *in vivo* animal experiment and stored at -80 °C were used to determine the levels of IFN-γ (Swine IFN-γ ELISA kit, Invitrogen, Ref. KSC4021), IL-10 (Swine IL-10 ELISA kit, Invitrogen, Ref. KSC0101) and TNF (Porcine TNF-alpha Quantikine ELISA Kit, R&D systems, Ref. PTA00), following the manufacturers’ instruction. These serum samples were also used to detect specific antibodies against the ASFV p72 protein using a commercial competition ELISA kit (INGEZIM PPA COMPAC, Ingenasa, Ref. 11.PPA.K3 Spain), following the manufacturer’s instructions.

Another set of equivalent aliquots containing frozen serum samples was treated at 56 °C for 30 minutes. The samples were then stored at -80 °C until they were analyzed. The level of cytokines and biochemical parameters were also evaluated in this set of heat-treated serum samples, as described above (section 2.3).

### 2.5 Statistical analysis

Statistical analysis and graphical representation of the data were performed using GraphPad Prism 10.01 (GraphPad Software, Inc., La Jolla, California, USA). A paired t-test was used to analyze differences in the concentrations of cytokines and biochemical analytes between heat-treated and untreated serum samples from domestic pigs used for routine laboratory diagnostics. Additionally, a paired t-test was used to analyze differences in white blood cell counts, cytokine concentrations and biochemical parameters between ASFV-infected animals that were euthanized on different days post-infection (day 1, 2, 3 and 5 pi, and at the humane endpoint) and their pre-infection values (day 0). These blood and serum parameters were also evaluated for differences in mock-infected control animals between days 0 and 8 after infection.

## 3 Results

### 3.1 Comparative evaluation of circulating white blood cells and antibody response in DP and WB infected with the ASFV “Armenia 2007” strain

**Figure 2:**
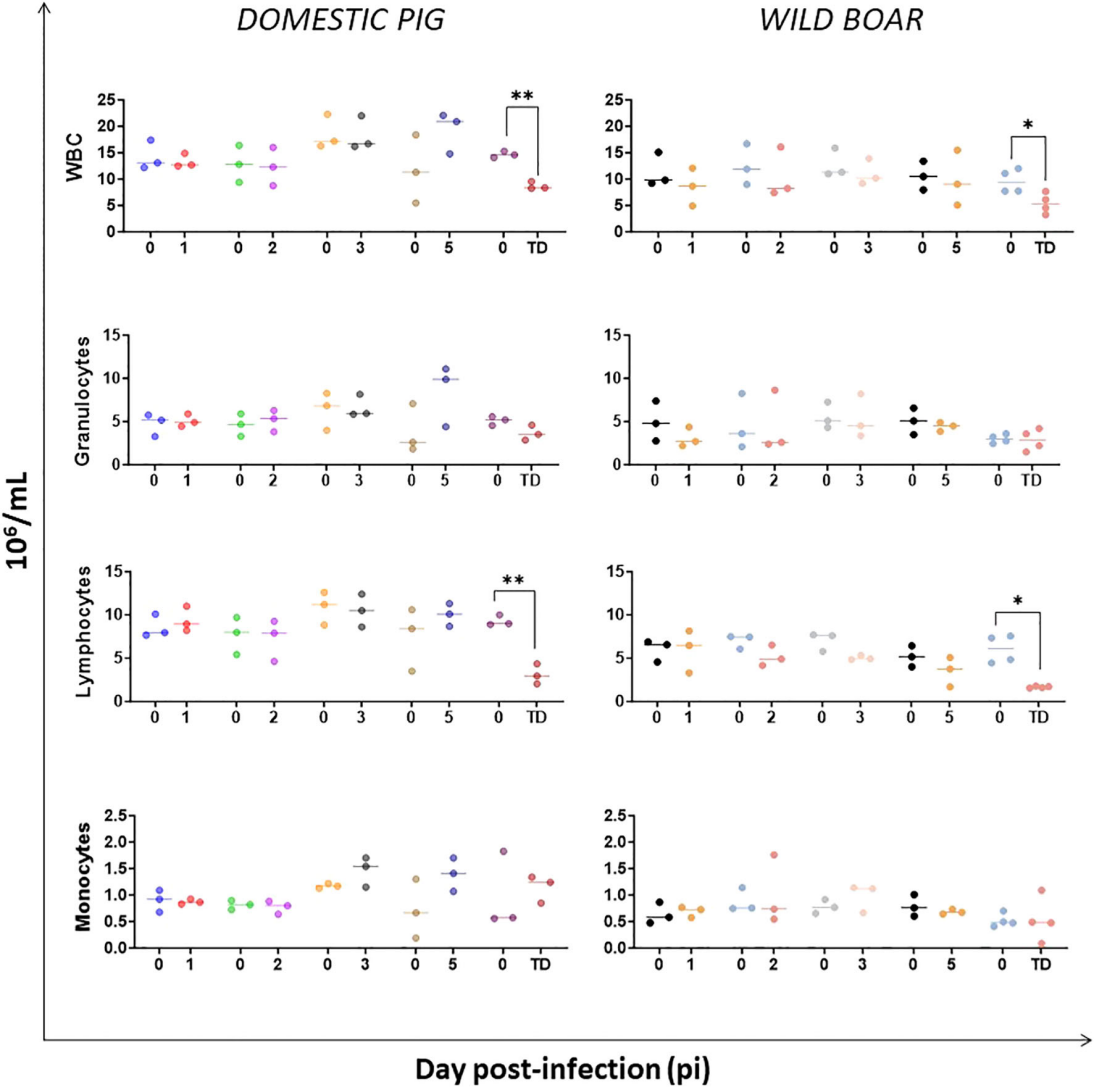
Leukocyte populations dynamics in domestic pigs and wild boar infected with ASFV genotype II strain “Armenia 2007”. Statistically significant differences (means) between days after infection with respect to their pre-inoculation values were evaluated using the paired t-test. Black asterisks indicate statistically significant differences. Day post-infection (x-axis); Number of cells per mL (y-axis); WBC: white blood cells (total number of leukocytes); TD: termination day (euthanasia was performed once the humane endpoint was reached); Variables of significance (*p ≤ 0.05; **p ≤ 0.01; ***p ≤ 0.001).

The levels of total leukocytes (WBC) in circulation were determined, including granulocytes, lymphocytes and monocytes. A significant decrease in the number of circulating total leukocytes, reflected by predominant and significant decrease in the number of lymphocytes, was observed in both infected DP (p=0.0040) and WB (p=0.0148) that were euthanized at the humane endpoint compared to pre-infection. It is worth noting the severe leukopenia and lymphopenia demonstrated by some of the wild boar (e.g. WB#53) from day 5 pi. Throughout the experiment, no changes in the subsets of granulocytes and monocytes were observed in infected animals (**Figure 2**).

Neither the infected DP nor the WB showed detectable levels of specific antibodies against ASFV in serum throughout the experiment based on cELISA evaluation.

### 3.2 Evaluation of cytokine dynamics in animals infected with the ASFV “Armenia 2007” strain

A preliminary evaluation of the impact of heat treatment on cytokine levels (IL-1α, IL-1β, IL-6, IL-8, IL-12, IL-18, and IL-1Ra) in serum samples from domestic pigs used for routine laboratory diagnostics revealed that heat treatment had no or minimal effect on the levels of these cytokines (**Supplementary Figure S1**). Based on these results, the cytokines were evaluated in heat-treated serum samples taken from DP and WB experimentally infected with the ASFV “Armenia 2007” strain. In addition, the cytokine study was conducted to evaluate TNF, IFN-γ and IL-10 levels in serum samples from infected DP and WB that had not been heat-treated.

**Figure 3:**
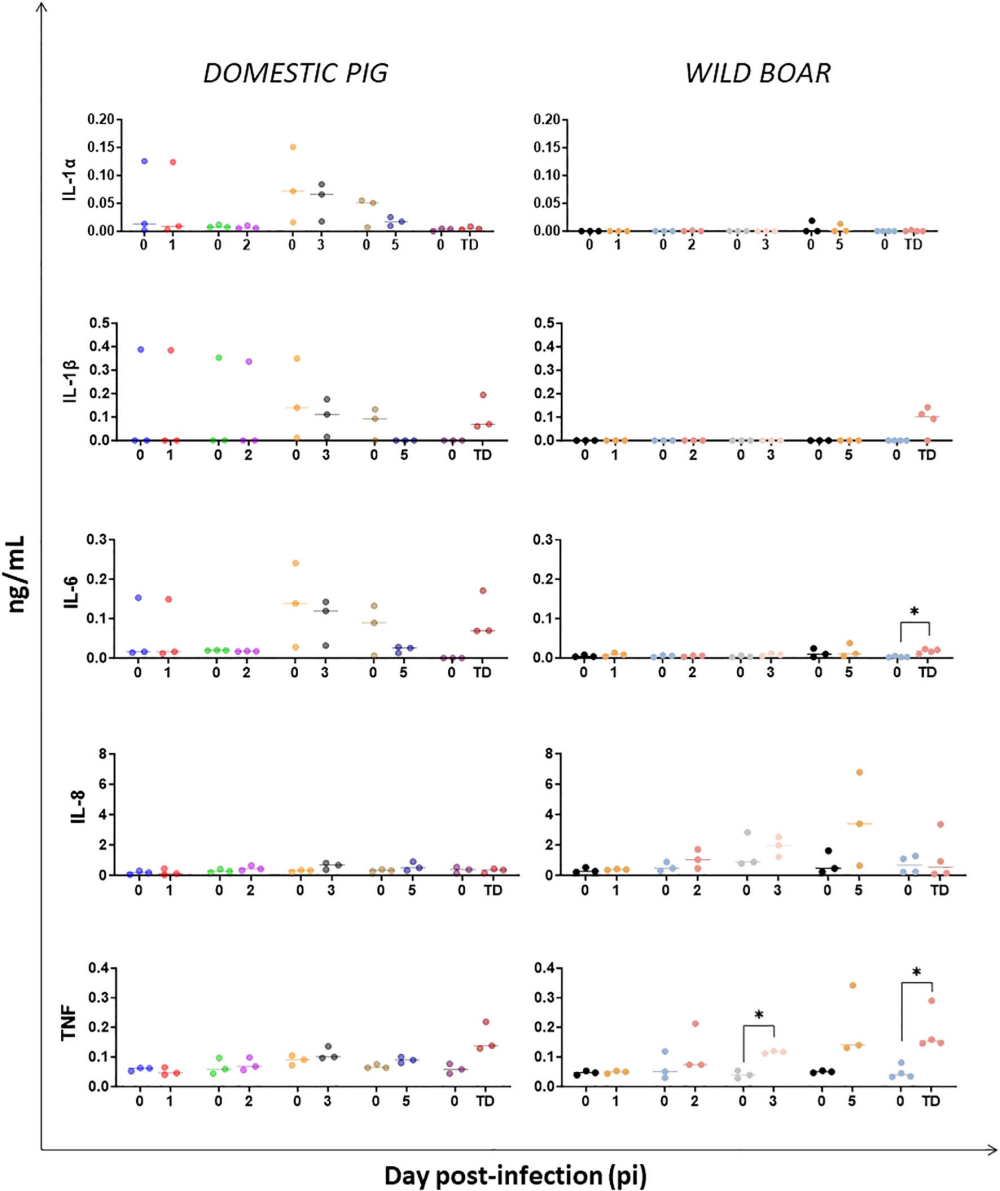
Evolution of serum levels of pro-inflammatory cytokines in domestic pigs and wild boar infected with ASFV genotype II strain “Armenia 2007”. Statistically significant differences (means) between days after infection with respect to their pre-inoculation values were evaluated using the paired t-test. Black asterisks indicate statistically significant differences. Day post-infection (x-axis); Cytokines concentration shown as ng/mL (y-axis); TD: termination day (euthanasia was performed once the humane endpoint was reached); Variables of significance (*p ≤ 0.05; **p ≤ 0.01; ***p ≤ 0.001).

Analysis of pro-inflammatory cytokines involved in immune regulation during the innate immune response revealed variable concentrations of IL-1α, IL-1β and IL-6 in DP prior to and during infection (**Figure 3**), though no significant changes from baseline were observed at any timepoints, except for IL-6 in WB at the humane endpoint. A rise in IL-1β was also noted in both groups at humane endpoint, although not significant. IL-8, which promotes neutrophil recruitment, increased over the course of infection in WB, but changes were not significant at any time-points in relation to pre-inoculation (**Figure 3**). However, a single WB (#53) exhibited the highest concentration of IL-8 at day 5 pi. For TNF, there was an increase in concentrations in infected animals, with a statistically significant increase observed earlier in WB from day 3 pi (p=0.015), and the highest levels being reached in WB (p=0.046) at the humane endpoint (**Figure 3**). Again, WB#53 had the highest concentration of TNF at day 5 pi. For DP, a rise in TNF serum levels was also noted at humane endpoint, although not significant.

**Figure 4:**
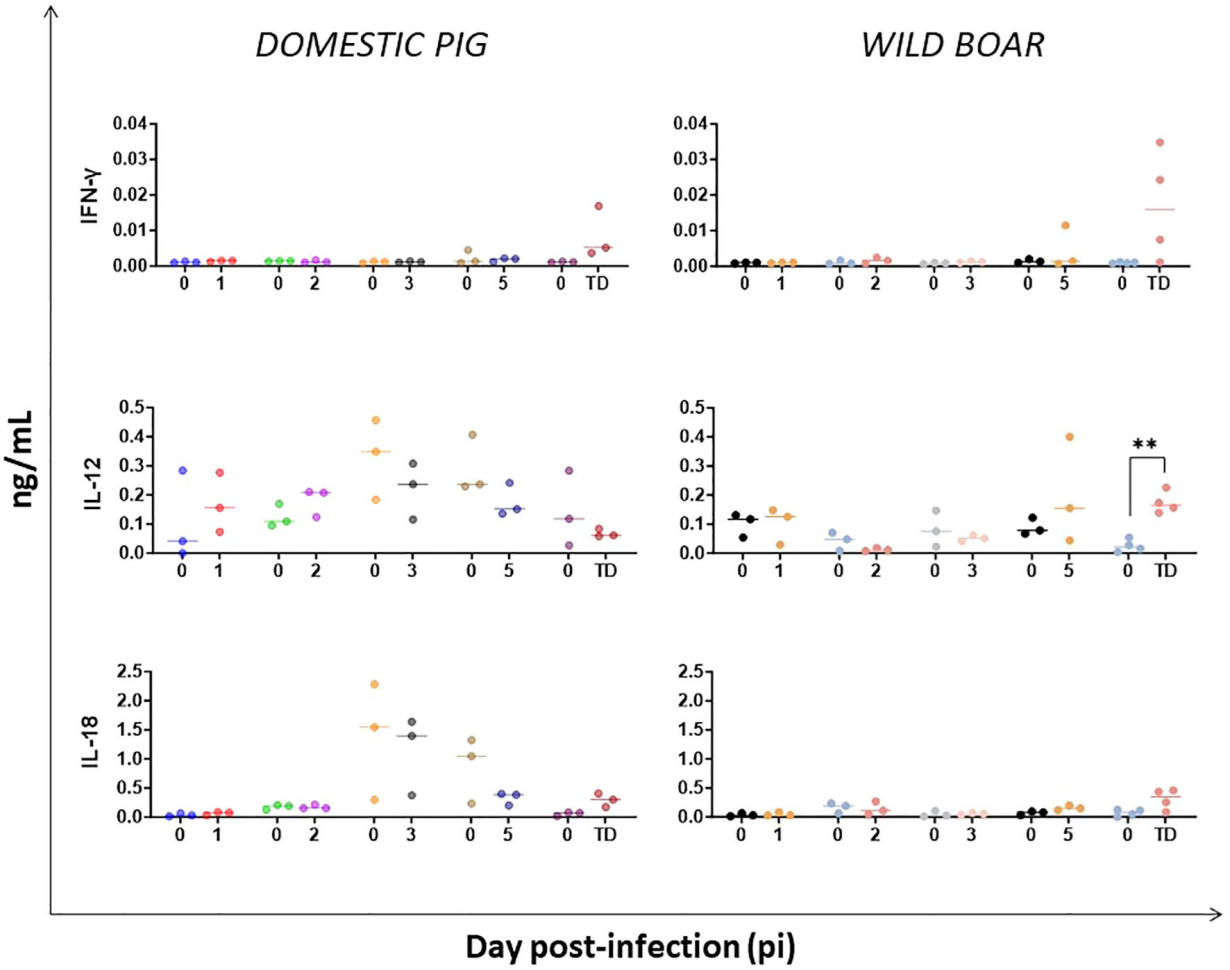
Evolution of serum levels of cytokines involved in Th-1 immune response in domestic pigs and wild boar infected with ASFV genotype II strain “Armenia 2007”. Statistically significant differences (means) between days after infection with respect to their pre-inoculation values were evaluated using the paired t-test. Black asterisks indicate statistically significant differences. Day post-infection (x-axis); Cytokines concentration shown as ng/mL (y-axis); TD: termination day (euthanasia was performed once the humane endpoint was reached); Variables of significance (*p ≤ 0.05; **p ≤ 0.01; ***p ≤ 0.001).

The serum concentrations of IFN-γ, IL-12 and IL-18 (**Figure 4**) were also evaluated to study the Th1 cytokine response. Throughout the experiment, there were no statistically significant changes in the serum concentrations of IFN-γ and IL-18 in either the infected DP or the WB. However, it is worth noting a gradual increase of IFN-γ concentrations at day 5 pi and at humane endpoint in both groups, including WB#53 at day 5 pi, although without significance. While substantial variations in IL-12 levels were observed in DP throughout the experiment, there was an increase in the infected WB from day 5 pi onwards, with WB#53 reaching the highest levels, and became significant (p=0.0099) at the humane endpoint (**Figure 4**).

**Figure 5:**
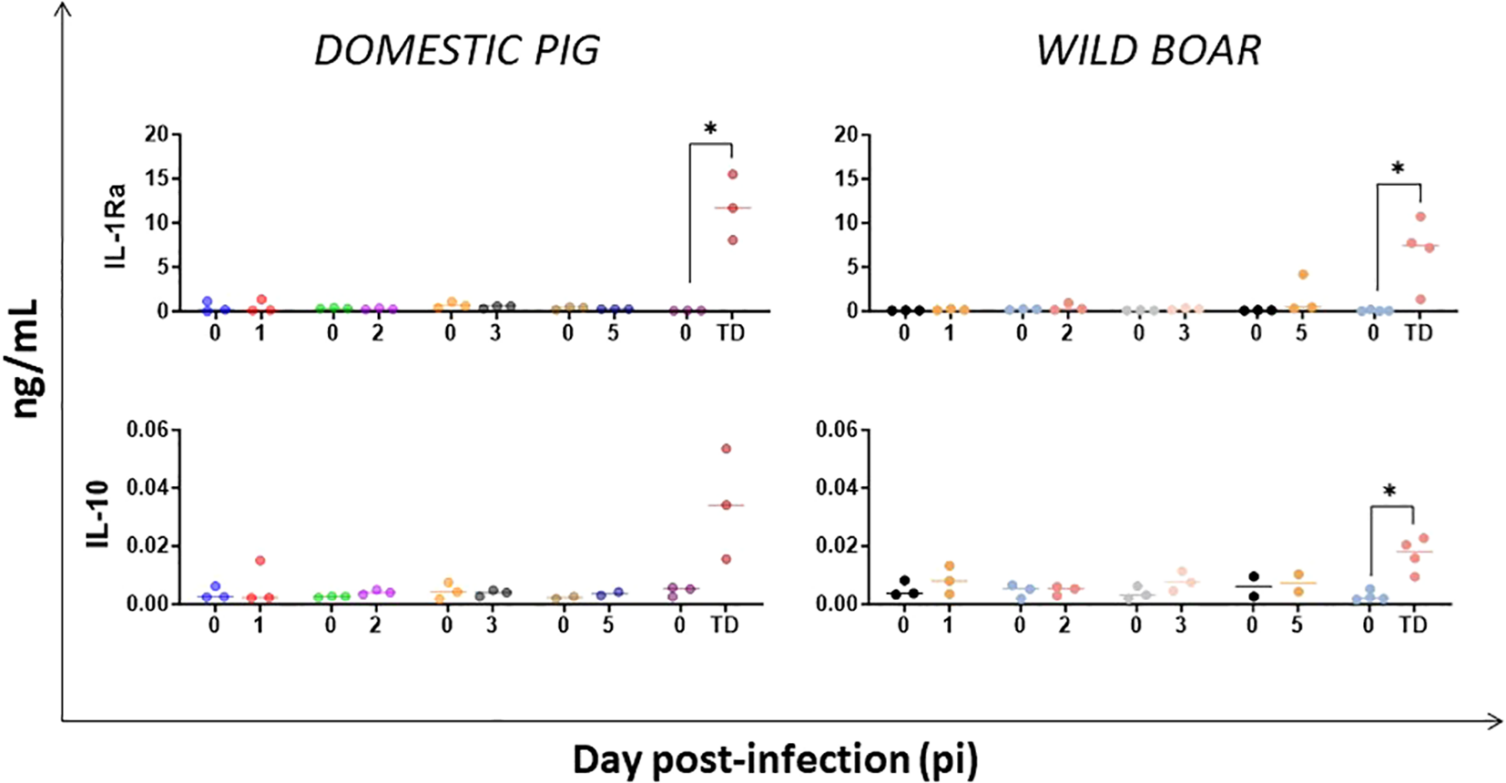
Evolution of serum levels of anti-inflammatory mediators in domestic pigs and wild boar infected with ASFV genotype II strain “Armenia 2007”. Statistically significant differences (means) between days after infection with respect to their pre-inoculation values were evaluated using the paired t-test. Black asterisks indicate statistically significant differences. Day post-infection (x-axis); Cytokines concentration shown as ng/mL (y-axis); TD: termination day (euthanasia was performed once the humane endpoint was reached); Variables of significance (*p ≤ 0.05; **p ≤ 0.01; ***p ≤ 0.001).

Serum concentrations of IL-1Ra (an IL-1 receptor antagonist involved in blocking the release of IL-1 and dampening inflammatory response) and IL-10 (an anti-inflammatory and immunosuppressive cytokine), were assessed. A delayed increase in the levels of IL-1Ra and IL-10 followed by a marked elevation by humane endpoint (p<0.05) was observed in both groups of infected animals (**Figure 5**).

### 3.3 Evaluation of sera biochemical profile of animals infected with the ASFV “Armenia 2007” strain

The heat treatment of serum samples from domestic pigs used for routine laboratory diagnostics was found to have little or no effect on C-reactive protein, AST, ALT, triglycerides, cholesterol, creatinine, calcium, urea, glucose, total protein, and albumin, as in humans (30). On the contrary, heat treatment significantly affected porcine serum CPK, ALP and GGT levels (see **Supplementary Figure S2**), which were not evaluated in heat-treated serum samples taken from DP and WB experimentally infected with the ASFV “Armenia 2007” strain.

**Figure 6:**
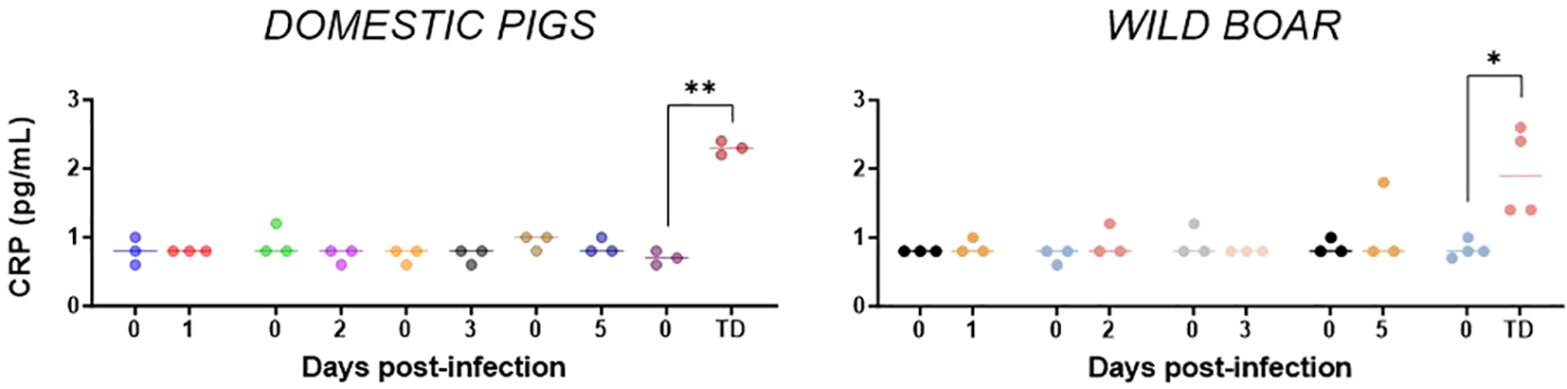
Evolution of C-reactive proteins in serum levels of domestic pigs and wild boar infected with ASFV genotype II strain “Armenia 2007”. Statistically significant differences (means) between days after infection with respect to their pre-inoculation values were evaluated using the paired t-test. Black asterisks indicate statistically significant differences. Day post-infection (x-axis); C-reactive concentration shown as pg/mL (y-axis); TD: termination day (euthanasia was performed once the humane endpoint was reached); Variables of significance (*p ≤ 0.05; **p ≤ 0.01; ***p ≤ 0.001).

C-reactive protein (CRP) is an acute-phase protein, which increases in response to inflammation, infection or injury. Our results revealed an increase in CRP concentrations in both groups, with some individuals within the WB group (e.g. WB#53) showing an increase by day 5 pi. CRP levels peaked at the humane endpoint in both groups, with concentrations significantly higher than pre-inoculation (p=0.0052 for DP and p=0.0454 for WB) (**Figure 6**).

**Figure 7:**
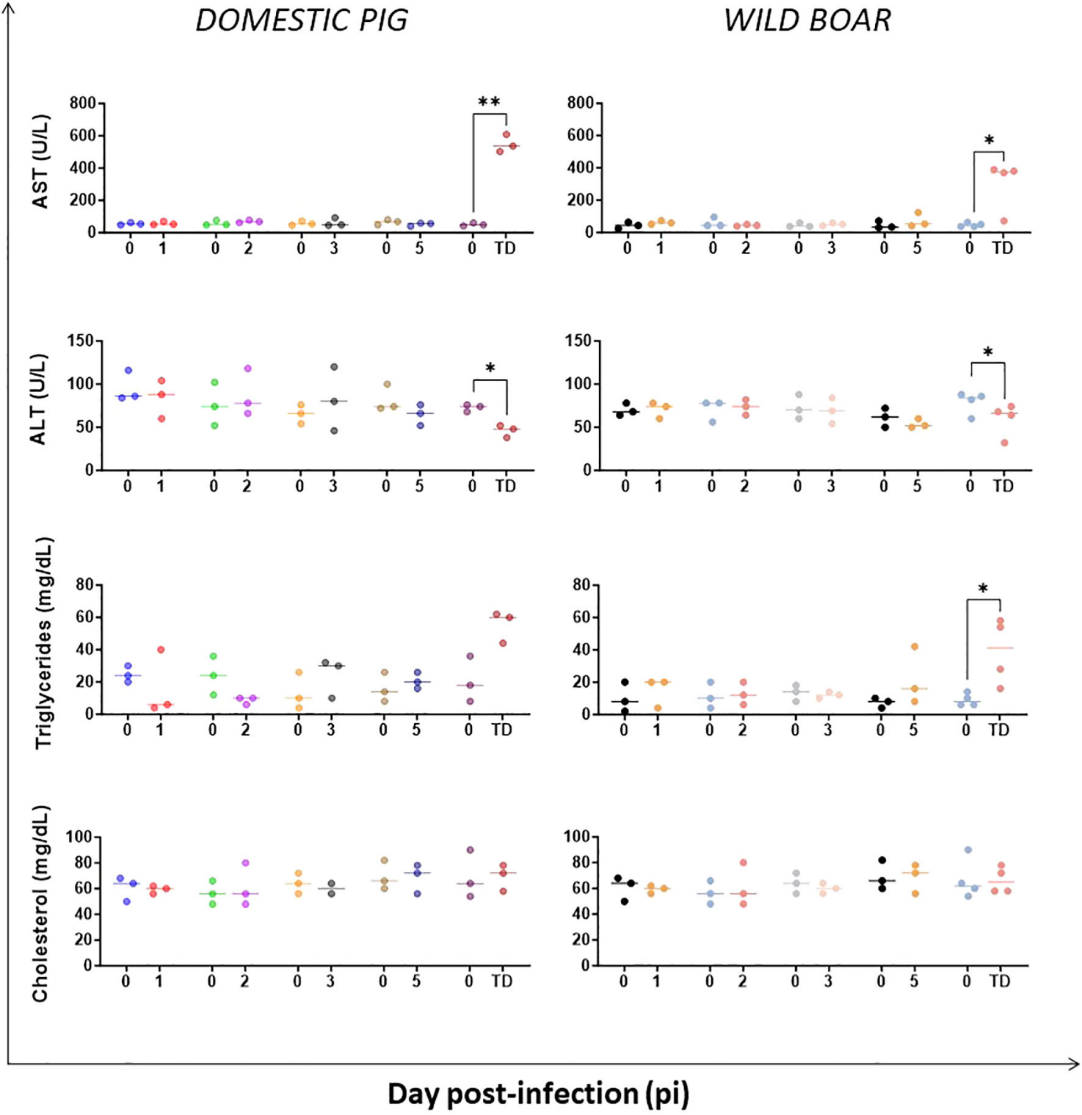
Evolution of serum concentrations of biochemical analytes indicative of liver function in domestic pigs and wild boar infected with ASFV genotype II strain “Armenia 2007”. Statistically significant differences (means) between days after infection with respect to their pre-inoculation values were evaluated using the paired t-test. Black asterisks indicate statistically significant differences. Day post-infection (x-axis); Concentration of analytes shown as mg/dL or Units/L (y-axis); TD: termination day (euthanasia was performed once the humane endpoint was reached); Variables of significance (*p ≤ 0.05; **p ≤ 0.01; ***p ≤ 0.001).

The concentrations of transaminases such as aspartate aminotransferase (AST) and alanine aminotransferase (ALT), indicators of liver injury, were assessed, as well as changes in serum triglyceride and cholesterol levels, which are also associated with liver damage or impairment. Biochemical analysis revealed a significant increase in serum AST and triglyceride levels compared to pre-inoculation levels at the humane endpoint in both groups, indicating a possible hepatic impairment (**Figure 7**). This was also evident in some individuals in the WB group (e.g. WB#53), which had high concentrations on day 5 pi. However, ALT levels were significantly lower than pre-infection values at the humane endpoint for both DP and WB, which could be related to the reduced appetite and food intake in these animals (33).

**Figure 8:**
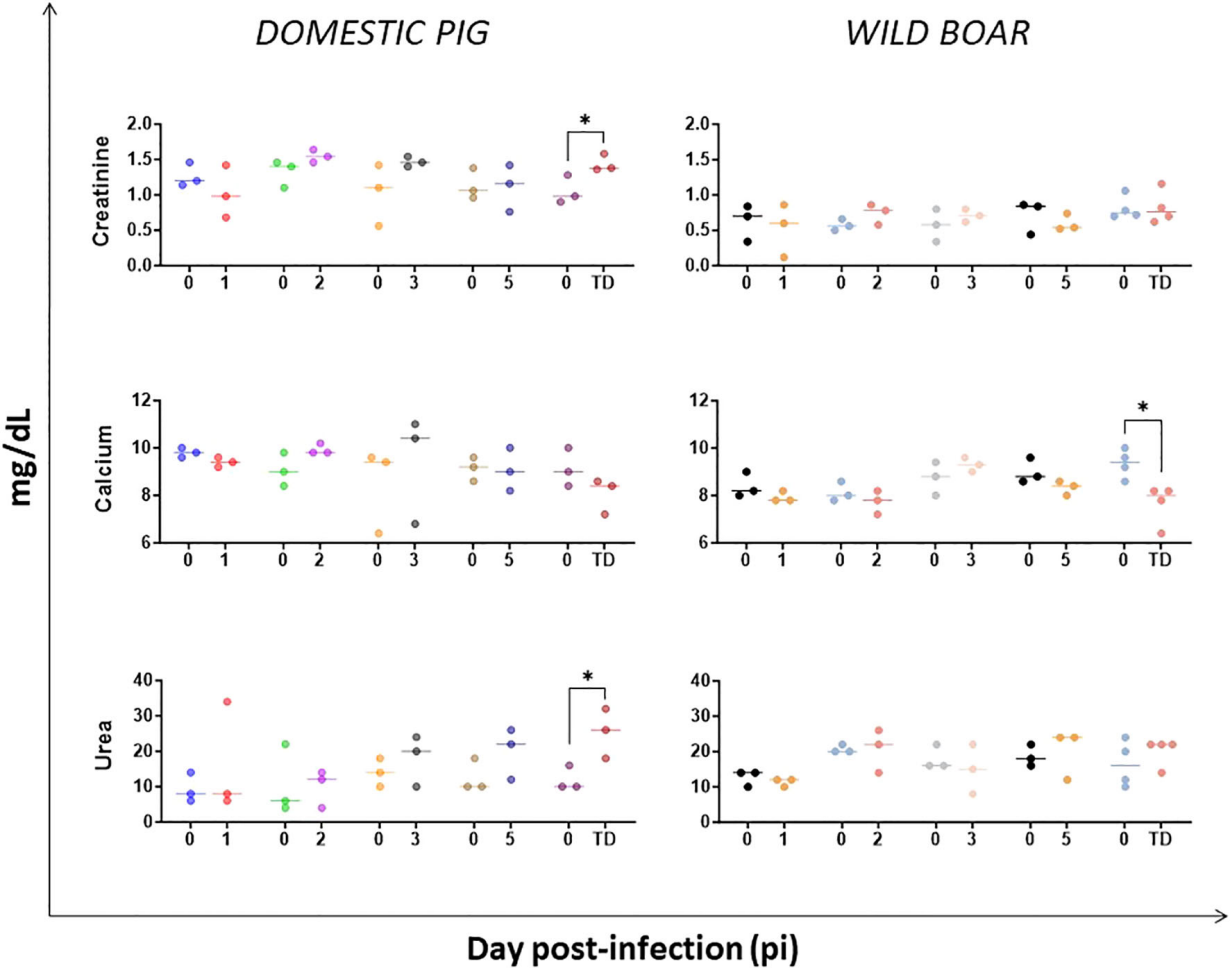
Evolution of serum concentrations of biochemical analytes indicative of renal function in domestic pigs and wild boar infected with ASFV genotype II strain “Armenia 2007”. Statistically significant differences (means) between days after infection with respect to their pre-inoculation values were evaluated using the paired t-test. Black asterisks indicate statistically significant differences. Day post-infection (x-axis); Concentration of analytes shown as mg/dL (y-axis); TD: termination day (euthanasia was performed once the humane endpoint was reached); Variables of significance (*p ≤ 0.05; **p ≤ 0.01; ***p ≤ 0.001).

**Figure 9:**
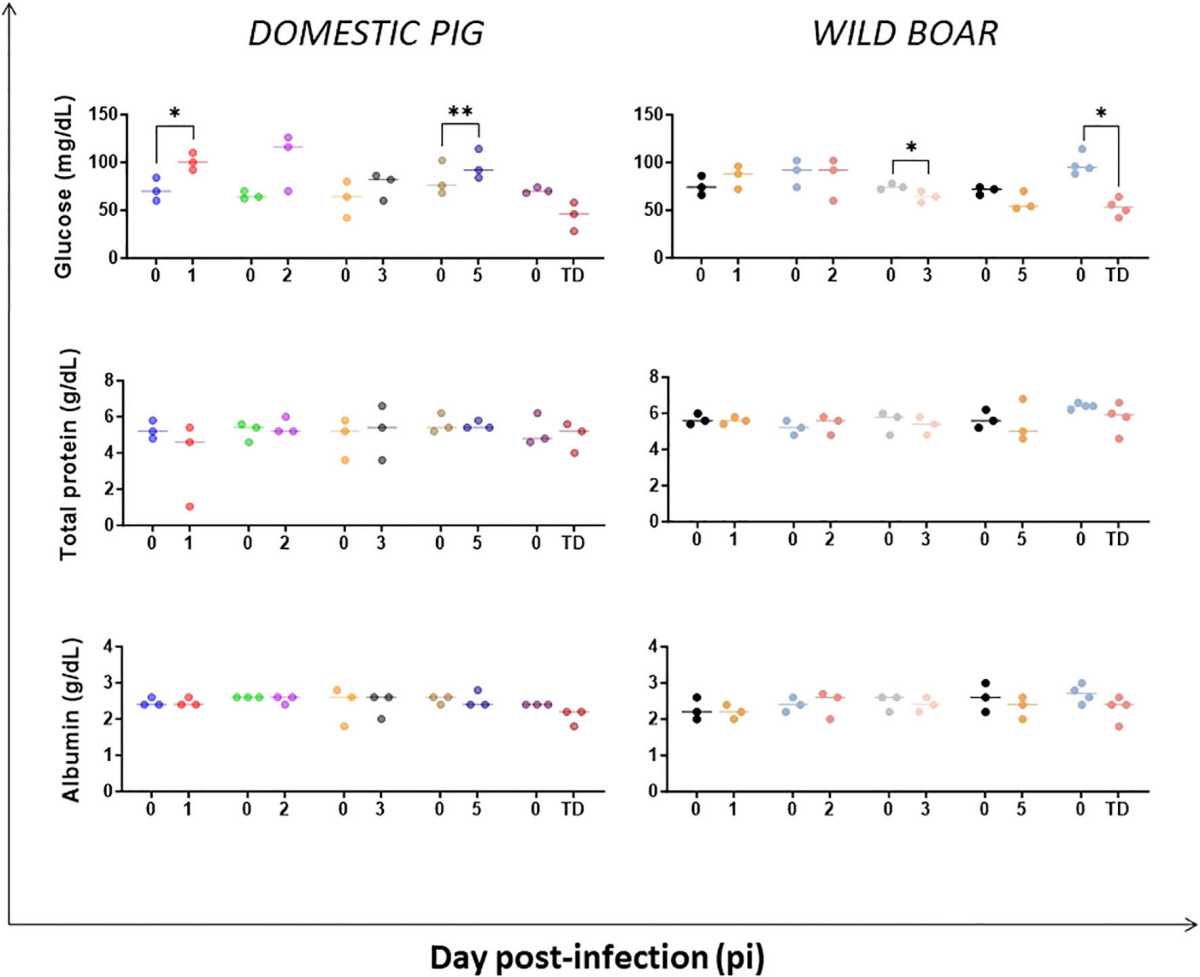
Evolution of serum concentrations of glucose, total proteins and albumin in domestic pigs and wild boar infected with ASFV genotype II strain “Armenia 2007”. Statistically significant differences (means) between days after infection with respect to their pre-inoculation values were evaluated using the paired t-test. Black asterisks indicate statistically significant differences. Day post-infection (x-axis); Concentration of analytes shown as mg/dL or mg/dL (y-axis); TD: termination day (euthanasia was performed once the humane endpoint was reached); Variables of significance (*p ≤ 0.05; **p ≤ 0.01; ***p ≤ 0.001).

We also evaluated the values of urea and creatinine, as well as electrolyte levels such as calcium, which are indicators of impaired renal function. Only the infected DP exhibited significant increases in urea and creatinine levels at the humane endpoint. However, calcium concentrations decreased in both groups at the humane endpoint, with this decrease being statistically significant in WB (**Figure 8**). In the absence of a significant reduction in protein levels (**Figure 9**) or overt gastrointestinal disease, a reduction in blood calcium could possibly suggest renal injury or potential derangement to the parathyroid regulation, of which the mechanism is unclear at present.

Albumin, total protein, and glucose levels were also monitored. There were no changes in serum albumin or total protein levels in either group throughout the experiment (**Figure 9**). Meanwhile, glucose levels fluctuated in the DP group and dropped at the humane endpoint in both experimental groups, with a moderate decrease also observed in WB at day 5 pi (**Figure 9**). A reduction of blood glucose could potentially account for some of the clinical signs observed in these animals, especially at the humane endpoint, including weakness, difficulty standing, lack of coordination, shakiness and tremors.

Finally, all blood and serum parameters were also monitored in mock-infected control animals at days 0 and 8 after infection. No differences between timepoints were observed in any of the evaluated parameters in these animals (**Supplementary Figure S3**).

## 4 Discussion

Our previous study demonstrated that WB were less resilient than DP following experimental intranasal infection with the ASFV “Armenia 2007” strain (27). The present study aims to gain an in-depth understanding of the mechanisms that govern immune system and biochemical dysregulation in these animals in the acute fatal forms of ASF, and to reveal any differences between DP and WB.

Intranasal infection with the virulent “Armenia 2007” strain resulted in severe lymphopenia, while monocyte and granulocyte levels remained unchanged throughout the experiment. The initial leukocytes profiles in both subspecies were comparable until day 5 pi, when some WB became lymphopenic, and most critically, lymphopenia coincided with fatal disease outcome at day 6 and 9 pi for WB and DP, respectively. Such correlation of haematologic and clinical outcomes for the DP were consistent with those of previous experimental studies with virulent genotype II ASFV strains regardless of the inoculation route (oronasal, intranasal or intramuscular) (25, 31, 34). Earlier lymphopenia (day 2–3 pi) has also been described in DP following intramuscular or intranasal infection with virulent ASFV strains belonging to genotype I (35) and II (36). However, early monocytopenia has only been observed following infection with virulent genotype I strains (35), but not with genotype II infections (24, 25, 31, 33), as in our experiment.

In contrast to our results, lymphopenia was not noted in experimental infections in WB with the virulent genotype II strain Armenia08 (25). These discrepancies may reflect genetic and immunological differences between WB and DP (37, 38), as well as host and viral factors such as the age of the animals, the virulence of the ASFV strains, or the doses and routes of inoculation. The macroscopic lesions previously described in the thymus (atrophy and hemorrhages) and lymph nodes (hemorrhagic lymphadenitis) of these DP and WB, especially at the humane endpoint (27), with the support of histopathological evidence of lymphoid depletion in the tonsils, lymph nodes, and spleen (39), were likely related to the lymphopenia described in the present study. As with acute ASF (40, 41), no specific antibodies against ASFV were detected in any of the infected animals due to the short interval between infection and the end of the study, as well as lymphoid depletion in the lymphorecticular organs (39), which would have impaired humoral immune responses.

TNF is a cytokine associated with fever, weight loss, and inflammation in response to infection (10, 42–44). Its early increase was identified as a key indicator of inflammatory response activation during our experiment, particularly in WB (from day 3 pi) in contrast to a later increase at the humane endpoint for DP. The early rise of TNF levels in WB was associated with the early increase in body temperatures and clinical signs from day 3–4 pi, contrasting to the clinical profile in DP which only appeared from day 7 pi onwards. Further, the early increase in serum TNF levels at day 3 dpi was was also associated with the onset of viremia and the detection of the virus in multiple tissues by qPCR and viral antigen by IHC (27, 39). Together, these findings provide a clear correlation between the onset of viral infection, activation of innate antiviral responses, and progression to clinical disease. Although there are conflicting results regarding changes in serum TNF levels following infection with virulent strains of ASFV, our results are consistent with those reporting a rapid increase in animals infected with the virulent genotype I (45, 46) and II strains (47, 48).

IL-6 concentrations increased in both DP and WB at humane endpoint, although only statistically significant in WB. A rise in IL-1β levels was also noted for both groups at humane endpoint. The rise of both IL-1β and IL-6 in DP and WB at humane endpoints coincided with the maximum body temperatures, clinical scores, viremia levels, viral loads in tissues and macroscopic lesion scores (27). These results are consistent with those of previous experimental infections involving virulent genotype I and II ASFV strains, which reported steady, high serum levels and maximum concentrations of these pro-inflammatory cytokines prior to death or at the humane endpoint. High levels of these cytokines have been linked to exaggerated, aberrant and failed activation of a protective immune response, with fatal consequences in ASFV infected pigs (40, 46–48), including vascular endothelial activation and damage (hemorrhages and oedema), lymphoid depletion, and immunosuppression (46, 49).

IL-8 (also known as CXCL8), which recruits and activates neutrophils, basophils, and some T-cells to sites of inflammation or infection (50), showed no significant changes in serum levels in DP, consistent with previous studies (51, 52). However, elevated concentrations of IL-8 were detected in some WB on day 5 pi and at the humane endpoint. As suggested in previous experimental infections with Armenia07 in pigs (41), IL-8 could potentially serve as an indicator of the manifestation of clinical signs and viremia. Both of these were certainly present in the WB group from day 5 pi onwards, but were delayed in the DP group (27).

IL-12 and IL-18, released mainly by antigen presenting cells, are key inducers of cell-mediated immunity, stimulating Th1 response and inducing IFN-γ production by NK cells and CD8+ cytotoxic T cells, which kill virus-infected cells (53). Consistent with our findings, previous studies reported no significant changes to these cytokines in DP during ASFV infection (54, 55). In contrast, sustained increases in the circulating levels of IL-12 and IL-18 were observed in DP inoculated intramuscularly with the virulent genotype II ASFV SY18 (48). Interestingly, significant high serum levels of IL-12 were detected in WB at the humane endpoint, and even as early as day 5 pi. This increase was also accompanied by a marked, albeit not statistically significant, rise in IFN-γ levels in the same animals, less evident in DP at humane endpoint, which is consistent with studies on ASF that reported elevated serum IFN-γ levels prior to death (40, 56, 57). Nevertheless, the late increase in serum IL-12 and IFN-γ levels in WB likely represents a failed attempt at disease protection, reflecting lower resilience to ASFV infection. An uncontrolled immunopathological response is less likely given the lower degree of macro- and microscopic tissue damage observed. This contrasts with DP, where IL-12 and IFN-γ peaked early (3 dpi) before viral dissemination, indicating an early protective attempt.

Consistent with previous studies, which reported high serum IL-10 and IL-1Ra in DP and WB infected with virulent genotypes I and II ASFV isolates (40, 48, 52, 55, 57–59), our results revealed a delayed increase in these mediators in both DP and WB, with an earlier increase seen in the WB group from day 5 pi. IL-10 is a potent anti-inflammatory and immune-suppressive cytokine released by several cell types, including macrophages, B cells, NK cells and T cell subsets, especially Treg cells (60), whereas IL-1Ra is a receptor antagonist, which is released to block IL-1 activity, preventing the development of inflammation and of an exacerbated immune response (43). In a separate study, Treg cells (one of the main cellular sources of IL-10) increased earlier and more frequently in WB than in DP, findings that correlated with greater disease severity and lethality in WB (25). Although an anti-inflammatory response was triggered in suids, it failed to curb the exaggerated and aberrant inflammatory response induced by ASFV infection, and as such, the late increase in IL-10 and IL-1Ra could be considered correlated negatively with animal survival.

CRP, a widely used marker for inflammation, is a major acute phase protein (APP) (61, 62). Our results revealed a significant increase in CRP concentrations in both groups of suids at humane endpoint, with some individuals within the WB group showing an increase at day 5 pi. The dynamics of CRP in blood samples, which increased from day 5 pi, were similar to those described in experimental infections with virulent genotype II strains (31, 34), although such increase was delayed compared to infections with genotype I strains (63). These differences could be attributed to the design of the experimental infections and the virulence of the virus strains. In any case, the CRP increase observed in some WB from day 5 pi was associated with increased IL-6, TNF, IL-12, and IL-8 concentrations on the same day. While IL-6 is well recognized as the primary driver of CRP production in the liver, IL-12 has also been shown to influence CRP levels and interact with other inflammatory markers, such as TNF, to induce CRP production (61, 64, 65). These immunologic interactions may be occurring in ASFV infected animals, including WB from day 5 pi. Furthermore, it should be noted that the increase in CRP levels from day 5 onwards, as detected in WB, would contribute to the induction of IL-8 (66). Therefore, hepatic lesion associated with viral infection in ASFV infected WB (27), along with pro-inflammatory cytokine induction, would contribute to the CRP synthesis and release (67).

Clinical biochemical analytes, infrequently assessed in ASFV studies, were assessed in this experiment. The concomitant increase of serum AST and triglyceride concentrations at humane endpoint were proxy of liver damage in DP and WB, with earlier changes in some WB presented with hepatic lesions (27). Previous studies in which domestic pigs were infected intranasally with different doses of a virulent Polish genotype II strain (Pol18_28298_O111) demonstrated an increase in AST serum levels but not ALT one to two days after the onset of viremia. The authors did not associate AST increase with liver failure, but rather with heart failure, despite the fact that inflammatory and degenerative changes described were more severe in the liver than in the heart of infected pigs (34). In contrast, an early increase in AST levels was reported in DP infected with a virulent Caucasian genotype II strain, which, as in our experiment, was associated with liver damage (68). However, in the same experiment, ALT concentrations, which are also associated with liver damage, increased moderately (68). These results were in contrast to the results obtained in our experiment, in which ALT levels decreased significantly in both DP and WB at the humane endpoint. Additionally, glucose levels declined in both groups, but earlier in WB group from day 5 pi. The reduction of both parameters could be associated with the reduced appetite and food intake observed in these animals (33), as well as the possibility of hepatic injury. A limitation of this study was the loss of CPK, ALP, and GGT data due to heat treatment, which otherwise would have provided useful insights into liver and cardiac disease.

The possibility of renal injury consequent to ASF infection was also examined given the inconsistency and sparsity of reports on renal biochemical profile and injury in the course of ASF infections. A significant increase in creatinine and urea levels, indicative of renal failure, was observed at the humane endpoint in DP but not in WB. Such differences could be attributed to less severe kidney lesions and circulatory disturbance in WB. Although ASFV antigen was detected in the kidney, no overt microscopic glomerular or tubular lesions were detected in WB (39). We hypothesize that the absence of functional renal impairment in WB may be due to the rapid clinical deterioration, which did not allow sufficient time for hemodynamic changes (e.g., hypovolemia) to compromise renal function. Alternatively, the disease course may have been too acute for biochemical markers to capture renal impairment. In other studies, no changes were observed in urea, creatinine, or calcium levels in DP infected intramuscularly with a virulent strain of genotype II (67), whereas intranasally challenged DP with a virulent strain of genotype II had a significant increase in creatinine and urea (34), suggesting possible influence over the disease pathogenesis by the route of virus inoculation.

Other notable biochemical derangement was hypocalcemia. Both DP and WB showed decreased blood calcium at the humane endpoint. This could indicate renal dysfunction (68), or alternatively an endocrine disturbance affecting parathyroid regulation. In a recent metabolomic study of sera of ASFV-infected pigs (69) enrichment of thyroid hormone signaling and autoimmune thyroid disease pathways was observed, suggesting possible involvement of thyroid-parathyroid function. It is also plausible that virus- or cytokine-mediated endocrine disruption led to reduced PTH secretion or cellular resistance. Other causes, such as impaired calcium absorption due to vitamin D deficiency from inappetence, could contribute but are more likely associated a more protracted disease progress. Further histopathological evaluation of thyroid and parathyroid glands is warranted, as these were not examined in our recent study (39).

Another limitation of this study that could influence the immunological profile is the age difference between cohorts (DP: 10–12 weeks; WB: 16–18 weeks), which reflects practical constraints in sourcing WB. Previous studies have reported even larger age gaps in similar *in vivo* experiments (3–20 months; 24). In DP, immune maturity stabilizes by approximately 8 weeks (70–72), and limited WB data suggest minimal immunological changes around 20 weeks (73). To balance these differences, we used slightly older WB compared to DP. ASFV studies indicate older pigs often have higher γδ/αβ T cell and NK cell levels, correlating with longer survival (54). If disease protection against ASFV were primarily age-dependent, WB would likely have survived longer, which was not observed. Thus, both cohorts were immunologically mature and similarly susceptible to ASFV. Differences in infection outcomes are unlikely to be explained solely by age and may reflect genetic and immunological factors (37, 38). However, intra-host factors remain an important knowledge gap for future research.

In conclusion, immunological, inflammatory and biochemical dysregulations occurred earlier in WB than in DP, underscoring the higher susceptibility or lower resiliency to disease in WB to ASFV infection. Both subspecies developed severe lymphopenia and concomitant inflammatory response, appearing earlier in WB. TNF increase was the earliest to be detected, followed by IL-6 and IL-1β, while IL-8 increased only in some WB. Th1-related cytokines IFN-γ and IL-12 rose moderately in WB from day 5 pi, whereas remained unchanged in DP, except of a little increase of IFN-γ at human endpoint, although without significance. CRP increased earlier in WB, and peaked in both groups at humane endpoint, alongside delayed increase in the levels of anti-inflammatory mediators IL-1Ra and IL-10. Biochemical analysis revealed potential perturbations of the liver function in both subspecies, characterized by changes in serum AST and triglycerides levels, in addition to renal alterations in DP evidenced by changes in creatinine and urea levels. Future research should aim to delineate molecular pathways at the tissue level and their relationship to systemic blood profiles, providing deeper mechanistic insights into ASFV pathogenesis and guiding strategies to mitigate disease impact across subspecies.

## Data Availability

The raw data supporting the conclusions of this article will be made available by the authors, without undue reservation.

## Supplementary material

Supplementary Figure 1 Impact of heat treatment on cytokine levels in serum samples collected from 30 domestic pigs undergoing routine diagnostic testing. Serum samples were divided into two groups. One set was left untreated, whereas the other was treated at 56 °C for 30 minutes. Treatment (x-axis); Cytokines concentration shown as ng/mL (y-axis).

Supplementary Figure 2 Impact of heat treatment on biochemical analytes in serum samples collected from 30 domestic pigs undergoing routine diagnostic testing. Serum samples were divided into two groups. One set was left untreated, whereas the other was treated at 56 °C for 30 minutes. Treatment (x-axis).

Supplementary Figure 3 Leukocyte populations, immune-regulatory cytokines, and biochemical parameters in control animals. Differences in leukocytes populations, C-reactive protein, pro-inflammatory cytokines (IL-1α, IL-1β, IL-6, IL-8, and TNF), anti-inflammatory mediators (IL-1Ra and IL-10), cytokines involved in Th-1 immune response (IFN-γ, IL-12 and IL-18), biochemical analytes indicative of liver function (AST, ALT, triglycerides and cholesterol), biochemical analytes indicative of renal function (urea, creatinine and calcium) and other analytes (glucose, total protein and albumin) in uninfected (controls) DP and WB between day 0 (before mock infection) and day 8 pi. Statistically significant differences were assessed using the paired t-test. Day post-infection (x-axis); Units of the parameter evaluated (y-axis); WBC: white blood cells (total number of leukocytes); DP: domestic pigs; WB: wild boar; Variables of significance (*p ≤ 0.05; **p ≤ 0.01).
