# Supplementary figures and images for: Dynamics of leukocyte populations, immune-regulatory cytokines, and biochemical parameters in wild boar and domestic pigs experimentally infected with a virulent African swine fever virus genotype II strain
Source: Front Immunol. 2026 Mar 24;17:1751646. doi: 10.3389/fimmu.2026.1751646 (PMC13054600; doi:10.3389/fimmu.2026.1751646)

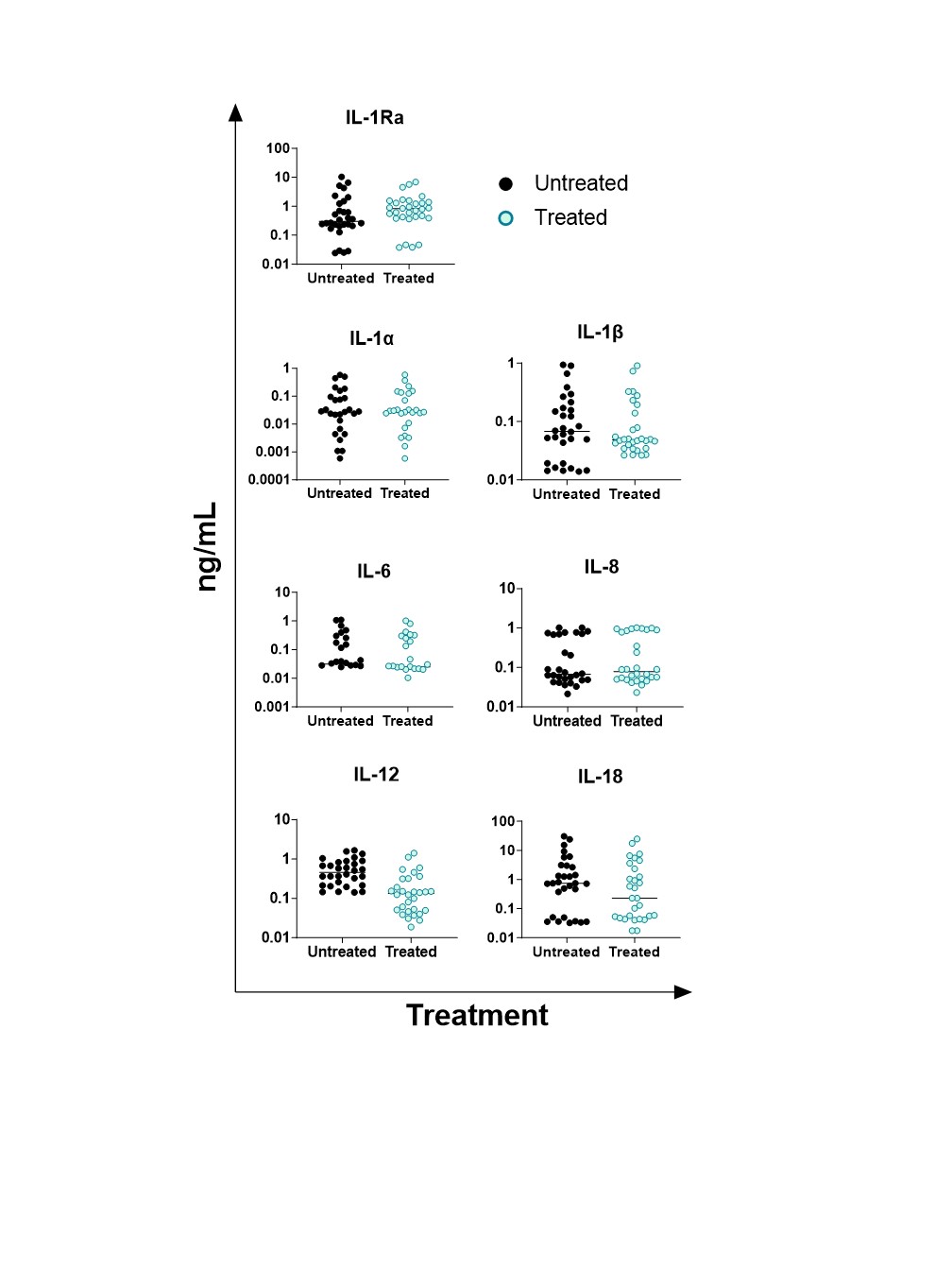

Supplement: Supplementary Figure 1 — Impact of heat treatment on cytokine levels in serum samples collected from 30 domestic pigs undergoing routine diagnostic testing. Serum samples were divided into two groups. One set was left untreated, whereas the other was treated at 56 °C for 30 minutes. Treatment (x-axis); Cytokines concentration shown as ng/mL (y-axis). [file Image1.jpeg]

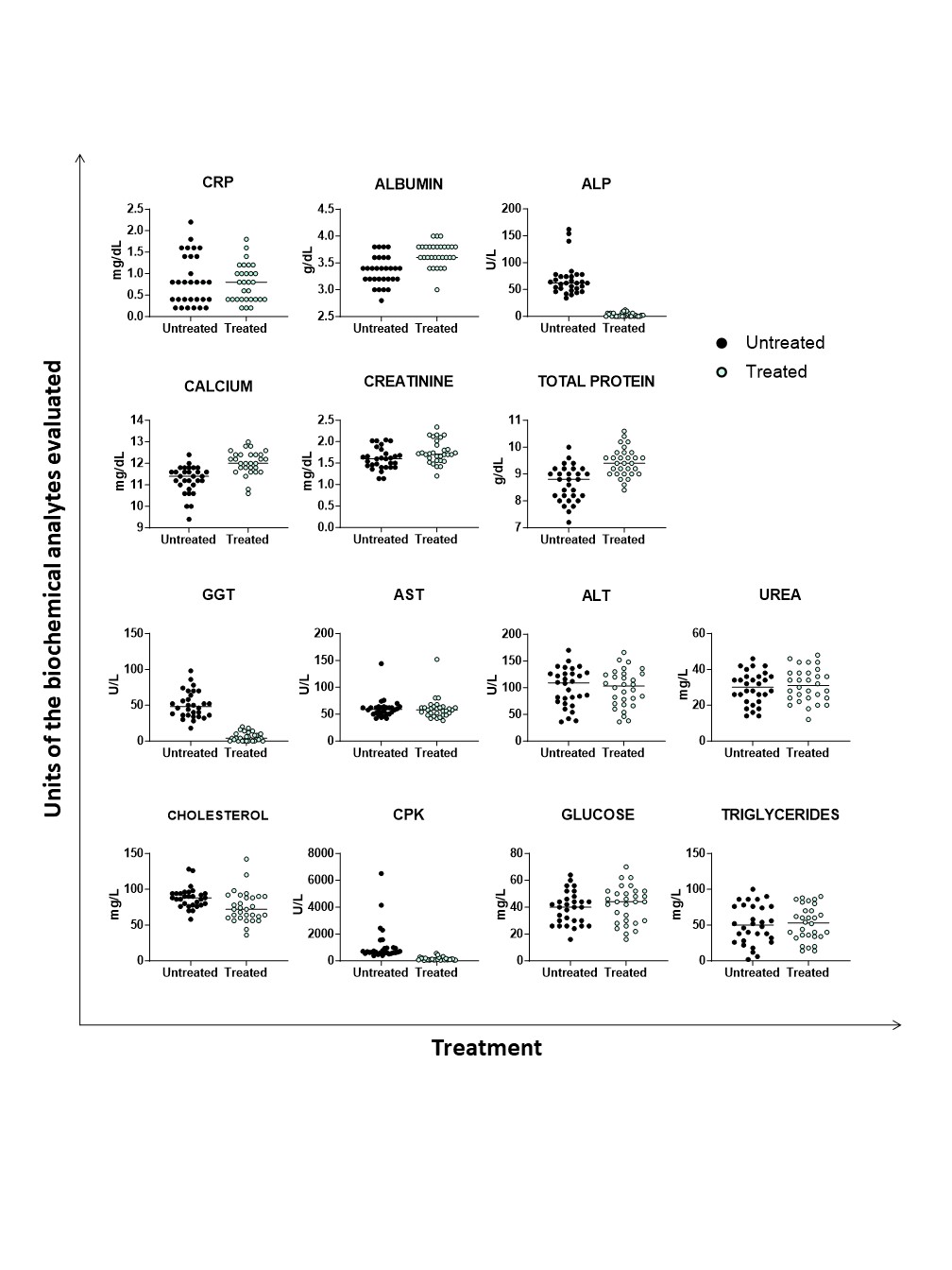

Supplement: Supplementary Figure 2 — Impact of heat treatment on biochemical analytes in serum samples collected from 30 domestic pigs undergoing routine diagnostic testing. Serum samples were divided into two groups. One set was left untreated, whereas the other was treated at 56 °C for 30 minutes. Treatment (x-axis). [file Image2.jpeg]

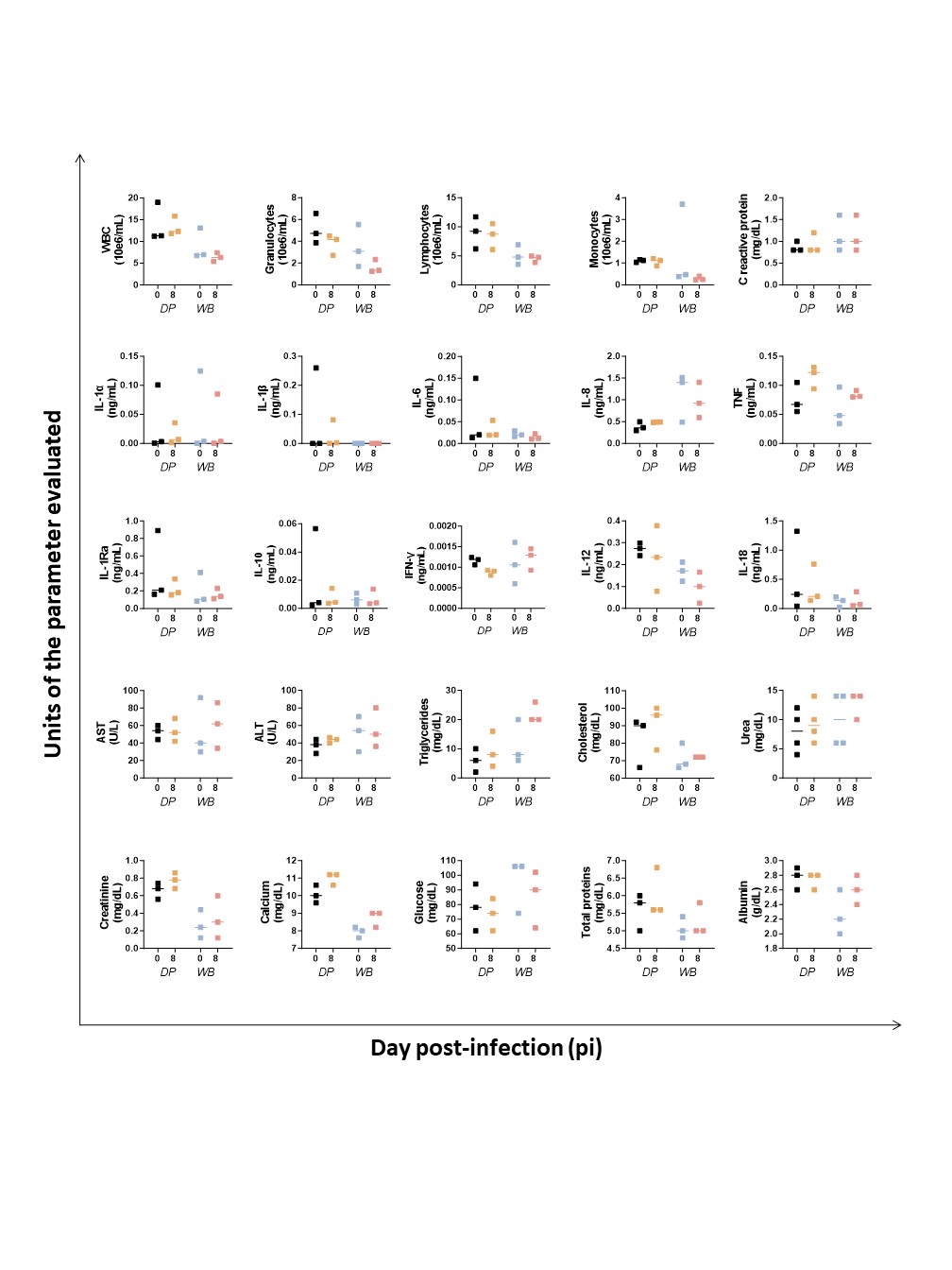

Supplement: Supplementary Figure 3 — Leukocyte populations, immune-regulatory cytokines, and biochemical parameters in control animals. Differences in leukocytes populations, C-reactive protein, pro-inflammatory cytokines (IL-1α, IL-1β, IL-6, IL-8, and TNF), anti-inflammatory mediators (IL-1Ra and IL-10), cytokines involved in Th-1 immune response (IFN-γ, IL-12 and IL-18), biochemical analytes indicative of liver function (AST, ALT, triglycerides and cholesterol), biochemical analytes indicative of renal function (urea, creatinine and calcium) and other analytes (glucose, total protein and albumin) in uninfected (controls) DP and WB between day 0 (before mock infection) and day 8 pi. Statistically significant differences were assessed using the paired t-test. Day post-infection (x-axis); Units of the parameter evaluated (y-axis); WBC: white blood cells (total number of leukocytes); DP: domestic pigs; WB: wild boar; Variables of significance (*p ≤ 0.05; **p ≤ 0.01). [file Image3.jpeg]
